# HYPR4D kernel method on TOF PET data with validations including image-derived input function

**DOI:** 10.1186/s40658-022-00507-6

**Published:** 2022-11-17

**Authors:** Ju-Chieh Kevin Cheng, Connor W. J. Bevington, Vesna Sossi

**Affiliations:** 1 Pacific Parkinson’s Research Centre, University of British Columbia, Vancouver, BC Canada; 2 Department of Physics and Astronomy, University of British Columbia, Vancouver, BC Canada

**Keywords:** HYPR4D kernel method, PSF, TOF PET, IDIF

## Abstract

**Background:**

Positron emission tomography (PET) images are typically noisy especially in dynamic imaging where the PET data are divided into a number of short temporal frames often with a low number of counts. As a result, image features such as contrast and time–activity curves are highly variable. Noise reduction in PET is thus essential. Typical noise reduction methods tend to not preserve image features/patterns (e.g. contrast and size dependent) accurately. In this work, we report the first application of our HYPR4D kernel method on time-of-flight (TOF) PET data (i.e. PSF-HYPR4D-K-TOFOSEM). The proposed HYPR4D kernel method makes use of the mean 4D high frequency features and inconsistent noise patterns over OSEM subsets as well as the low noise property of the early reconstruction updates to achieve prior-free de-noising. The method was implemented and tested on the GE SIGNA PET/MR and was compared to the TOF reconstructions with PSF resolution modeling available on the system, namely PSF-TOFOSEM with and without standard post filter and PSF-TOFBSREM (TOF Q.Clear) with various beta values (regularization strengths).

**Results:**

Results from experimental contrast phantom and human subject data with various PET tracers showed that the proposed method provides more robust and accurate image features compared to other regularization methods. The preservation of contrast for the PSF-HYPR4D-K-TOFOSEM was observed to be better and less dependent on the contrast and size of the target structures as compared to TOF Q.Clear and PSF-TOFOSEM with filter. At the same contrast level, PSF-HYPR4D-K-TOFOSEM achieved better 4D noise suppression than other methods (e.g. >2 times lower noise than TOF Q.Clear at the highest contrast). We also present a novel voxel search method to obtain an image-derived input function (IDIF) and demonstrate that the obtained IDIF is the most quantitative w.r.t. the measured blood samples when the acquired data are reconstructed with PSF-HYPR4D-K-TOFOSEM.

**Conclusions:**

The overall results support superior performance of the PSF-HYPR4D-K-TOFOSEM for TOF PET data and demonstrate that the proposed method is likely suitable for all imaging tasks including the generation of IDIF without requiring any prior information as well as further improving the effective sensitivity of the imaging system.

## Introduction

Positron emission tomography (PET) images are typically noisy especially in dynamic imaging where the PET data are divided into a number of short temporal frames often with a low number of counts. As a result, image features such as contrast and time–activity curves (TACs) are highly variable. Therefore, noise reduction in PET is essential. Typical noise reduction methods reduce noise at the cost of reduced contrast or accuracy; i.e. they tend not to preserve image features/patterns accurately (e.g. accuracy is contrast and size dependent). In dynamic PET, target structures of interest have various sizes with different contrasts which vary over time at different rates. Consequently, a noise reduction method that can preserve image features independently of the contrast and size of the structure is highly desirable.

Inspired by kernel methods in machine learning, kernelized reconstruction has shown promising results in PET de-noising while preserving contrast [1, 2]. Kernelized reconstruction methods typically reparametrize the image estimate (*λ*) into a kernel matrix (K), which contains an alternative set of basis functions with feature vectors extracted from guiding image(s), and a vector of kernel coefficients (α) which can be viewed as a latent representation of the image estimate. Consequently, each image voxel intensity is modeled as a function of a set of (desirable) features obtained from the guiding image(s). Most kernelized reconstructions utilize the Non-Local Mean (NLM) [3] kernel by looking at the similarity between voxels or patches of voxels in the guiding image(s); the more recent kernel method based on HighlY constrained backPRojection (HYPR) [4], which makes use of the high frequency features in the guiding image(s), has been reported to outperform the NLM kernel in terms of implementation simplicity and noise reduction performance [5].

In conventional kernel methods, high signal-to-noise ratio guiding image(s), from which spatial intensity based feature vectors are extracted, are typically pre-defined using either anatomical MRI or the temporal sum of PET data. As a result, the kernel matrix contains very little or no temporal information on PET tracers’ distribution and is thus unable to provide sufficient temporal noise reduction nor properly ‘track’ and preserve the temporal pattern of PET tracers. Moreover, the pre-defined guiding image(s) can introduce bias into PET images whenever there are mismatches in features between guiding image(s) and target PET images [2].

Recently, a spatiotemporal kernel method has been proposed to achieve high temporal resolution (HTR) by incorporating a single temporal kernel extracted from PET sinogram data [6]. More recently, we have proposed an intrinsic data-driven/prior-free 4D kernel method based on 4D modified HYPR (i.e. HYPR4D). It utilizes a truly 4D feature vector which applies voxel-specific temporal kernels generated directly within the reconstruction. It has been demonstrated to have better preservation of spatiotemporal patterns while achieving 4D noise reduction as compared to the HTR kernel method and other standard noise reduction methods on conventional non-Time Of Flight (TOF) PET data [7].

In this work, we implemented our HYPR4D kernel method to reconstruct TOF PET data (i.e. PSF-HYPR4D-K-TOFOSEM) acquired on the GE SIGNA PET/MR system which has a TOF resolution of ~400ps [8]. We compared its performance in terms of contrast recovery and noise suppression as well as quality of time–activity curves from relatively small structures to the performance achieved with all the available TOF reconstructions with point-spread-function (PSF) resolution modeling on the system, namely PSF-TOFOSEM with and without standard (3.5mm FWHM transaxial and 1-4-1 axial) post filter and PSF-TOFBSREM (i.e. TOF Q.Clear which is the newly introduced state-of-the-art clinical reconstruction method) [9, 10] with various beta values (regularization strengths) using data acquired from contrast phantom and human subjects. In addition, the impact of the reconstruction method on the derivation of an image-derived input function (IDIF) obtained from a novel voxel search technique was investigated. For a more extensive comparison of the HYPR4D kernel method with other image reconstruction methods without resolution modeling for non-TOF data, readers are referred to [7].

## Materials and methods

### PSF-HYPR4D-K-TOFOSEM

The HYPR4D kernel matrix consists of basis functions using spatiotemporally variant convolution (constructed based on the guiding image). The guiding image is computed as the sum of de-noised subset estimates from the previous iteration which can be generated directly within the reconstruction at every time point. As a result, a truly 4D and purely data-driven (high spatiotemporal frequency) feature vector can be obtained. The proposed PSF-HYPR4D-K-TOFOSEM is given by:

1 $${\alpha }_{4D}^{m,s}=\frac{{\alpha }_{4D}^{m,s-1}}{{({K}_{H4D}^{m})}^{T}{P}_{s;l;t}^{T}1}\cdot \left({({K}_{H4D}^{m})}^{T}{P}_{s;l;t}^{T}\frac{{y}_{4D}^{s;l;t}}{{P}_{s;l;t}{K}_{H4D}^{m}{\alpha }_{4D}^{m,s-1}+{b}_{4D}^{s;l;t}}\right)$$

2 $${\lambda }_{4D}^{m,s}={K}_{H4D}^{m}{\alpha }_{4D}^{m,s}$$

3 $${K}_{H4D}^{m}={\mathrm{diag}}\left[{h}^{m}\right]{F}_{4D},\quad {\mathrm{where}}\; {h}^{m}=\frac{{C}_{4D}^{m}}{{F}_{4D}*{C}_{4D}^{m}}$$

4 $${C}_{4D}^{m}=\sum_{s}{K}_{H4D}^{m-1}{\alpha }_{4D}^{m-1,s}$$

where *α*_*4D*_^*m,s*^ is the 4D kernel coefficient at *s*th subset of *m*th iteration, *K*_*H4D*_^*m*^ is the HYPR4D kernel matrix which is decomposed into the self-normalized spatiotemporal weights extracted from the 4D guiding image (*C*_*4D*_^*m*^) for the preservation of 4D high frequency features (*h*^*m*^) and the spatiotemporally invariant 4D Gaussian convolution (*F*_*4D*_). The sparsity of the kernel matrix only depends on the width of the 4D Gaussian since the matrix which contains *h*^*m*^ is diagonal. *P*_*s;l;t*_ is the system matrix for the *t*th TOF bin along the *l*th line-of-response (LOR) within the *s*th subset; the sinogram/projection-based resolution modeling with spatially variant PSF and time spread function used for TOF reconstruction are embedded here along with normalization and attenuation corrections. *y*_*4D*_^*s;l;t*^ is the measured dynamic 4D TOF sinogram data, *b*_*4D*_^*s;l;t*^ is the estimate of background contamination such as randoms and scattered coincidences at *t*th TOF bin along the *l*th LOR within the *s*th subset, and *λ*_*4D*_^*m,s*^ is the 4D (de-noised) PET image estimate at *s*th subset of *m*th iteration. For the GE SIGNA PET/MR implementation presented in this work, *s* = 1, …, 28, *l* = 1, …, 5657736 (i.e. 357*224*1981/28), and *t* = 1, …, 27. Note that in TOF reconstructions, data events/counts are assigned to each TOF bin, and the forward- and back-projections are performed within each TOF bin instead of the whole LOR.

One iteration of PSF-TOFOSEM was used to initialize the 4D guiding image (i.e. sum of subset updates within the first iteration of PSF-TOFOSEM) in the kernel matrix. The one PSF-TOFOSEM iteration images are also used as the input initial 4D estimate for Eq. (1). After the 1st HYPR4D iteration, the guiding image is updated using the de-noised subset estimates from the previous iteration as shown in Eq. (4) and thus provides a highly constrained noise increment per update and allows the 4D high frequency features to be updated in a cleaner data-driven fashion as compared to conventional methods.

Note that in the sinogram/projection-based reconstruction, different OSEM subset data correspond to different realizations of the same tracer distribution observed from different angular views; they do not necessarily agree with one another especially at low count situations. As a result, limit cycle behavior or oscillation in values of image features, such as contrast recovery, over image estimates derived from different subsets is typically observed. Additionally, the standard OSEM-based final image estimate is biased toward the data from the last OSEM subset as the latter is used in the final update. On the other hand, the OSEM subset data and corresponding image estimates also do not share the same noise pattern. Consequently, by summing the images obtained from each subset, the true signals exhibit mostly constructive interference unlike noise. Therefore, the high frequency features extracted from the guiding image/sum over the OSEM subset estimates (i.e. the mean high frequency features) contain more information about structure boundaries and less so about noise than those obtained from each individual subset estimate. They are thus used to discourage the inconsistent image features over the OSEM subset estimates in the proposed method.

In short, the proposed method makes use of (i) inconsistent noise patterns over the OSEM subsets to minimize noise, (ii) the mean high frequency features over the subset data/extracted from the guiding image to regulate the subset features, and (iii) the low noise property of early updates of the reconstruction to achieve prior-free noise constraint/reduction. The progressive update of the 4D guiding image ensures the extracted 4D high frequency features are adaptive to the measured PET data. As a result, better preservation of spatiotemporal patterns can be attained by the proposed method as compared to other methods while achieving 4D noise reduction. Additional benefits of the proposed method include reduction of (i) zero trapping (see the reduction of ‘holes’ when comparing the PSF-HYPR4D-K-TOFOSEM image to the PSF-TOFOSEM image in Fig. 4 top for an example), (ii) negative voxel contrast recovery coefficient as will be reported in the results, and (iii) limit cycle behaviour and bias towards the last OSEM subset data typically observed from OSEM reconstructions [7].

### Extracting image-derived input function (IDIF)

**Fig. 1:**
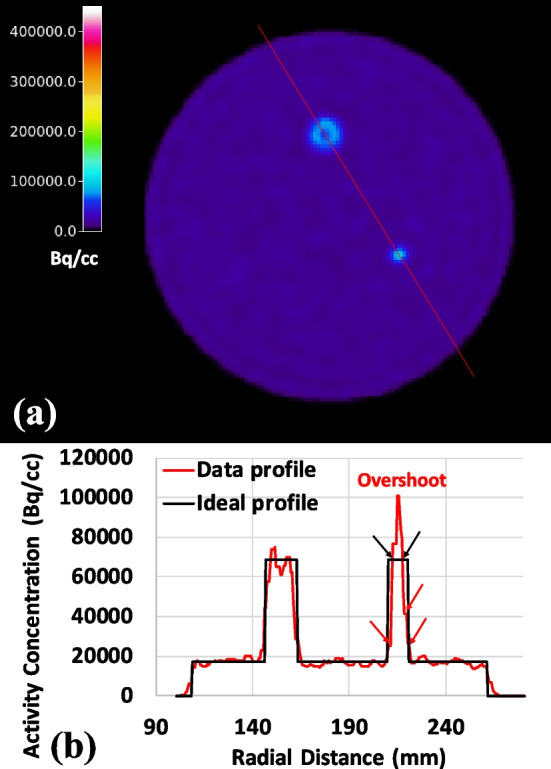
Illustration of Gibb’s artifact. **a** The transaxial view of a contrast phantom (consisting of two spheres with 17 mm and 10 mm in diameter and a 4:1 hot-to-background contrast ratio) reconstructed with PSF resolution modeling; Gibb’s artifact appears as a ‘ring’ in relatively big structures (e.g. 17 mm sphere) while the ring gets pushed toward the center of the structure and forms the overshoot when the structure is small enough (e.g. 10 mm or smaller) and **b** the corresponding line profile as shown in (**a**) with black arrows pointing at the locations with the correct activity concentration and red arrows pointing at the locations of underestimated voxels near the edge of the structure. Note that when using a volume-of-interest (VOI) with the physical dimension of the 10 mm sphere, a ~ 60% contrast recovery coefficient is obtained as shown in Fig. 2 since the underestimation near the edge of the structure outweighs the overestimation near the center of the structure

Image-derived input function may provide a very useful alternative to plasma input/blood sampling methods for quantification of selected tracer data. Several approaches have been proposed [11], but most still require an explicit partial volume correction due to the relatively small size of the carotid artery. In this work, we propose a voxel search method with a SpatioTemporal Constraint (STC) to find voxels within the image of carotids that are least affected by the partial volume effect (PVE), minimally impacted by Gibb’s artifacts [12] (see Fig. 1), and are best representative of the blood input function.

The proposed voxel search method for extracting PVE-minimized IDIF builds on the de-noising and preservation of spatiotemporal features of the proposed HYPR4D kernel method which enables the use of a very limited number of voxels to obtain the IDIF. As a result, strict voxel selection criteria can be applied. These criteria include: (i) minimizing the number of voxels with underestimated activity concentration within the carotids by correcting or rejecting them through the use of spatially variant PSF resolution modeling, Maximum Intensity Projection (MIP), and a spatial mask/constraint, (ii) applying a temporal constraint to reduce the contribution from surrounding tissues and noise to the IDIF peak timing, and (iii) rejecting voxels with overestimated activity concentration due to overshooting/Gibb’s artifacts as well as contamination from surrounding tissues (which can produce both overestimated and underestimated voxels) using three venous blood samples collected during the later half of the scan. [^18^F]FDG images were selected for implementation and testing of the method due to the practical relevance of an IDIF for this tracer.

Although the PSF resolution modeling applied within the reconstruction improves image voxel quantification within target structures, it does not completely correct for the PVE; e.g. activity concentration in voxels near structure boundaries tends to still be underestimated when the structure contains higher concentration than the surrounding tissues as depicted by the red arrows in Fig. 1b. To further reduce PVE, we propose a MIP-based voxel search. In this work, we are focusing on the carotid artery; i.e. a high-intensity small/narrow structure surrounded by low-intensity voxels during the early part of the scan. The fraction of voxels affected by PVE and the severity of PVE within the small structure depend on the effective resolution of the PET scanner (a combination of detector crystal size, positron range, reconstruction, filtering, etc.). When the size of the structure is close to the effective resolution of the scanner, nearly all voxels within the structure are affected by PVE, and the edge voxels are the most affected. The inclusion of an accurate PSF resolution model within the reconstruction introduces both overestimation and underestimation of voxel values within the small structure. As illustrated in Fig. 1, the underestimation near the edge of the relatively small (10mm) structure is worse than the overestimation near the center. The MIP voxels generally contain those central voxels within the high-intensity small structures after intensity thresholding. This approach defines our spatial constraint without the need of an accurate structural segmentation (as long as there is no other structure with higher intensity voxels along the projection axis). Our voxel search method then uses the measured blood samples to reject the voxels with overly overestimated/underestimated activity concentration.

By examining the radioactivity spatial distribution in images obtained from several tracers (e.g. [^11^C]RAC, [^11^C]DTBZ, and [^18^F]FDG) acquired within the first minute, it was found that the sagittal MIP contains lower occurrences of overlapping signals between the carotid arteries and veins/sinuses along the projection axis compared to transverse and coronal MIPs. Therefore, the sagittal MIP was used for the proposed voxel search method. Reconstructed dynamic images were split into the left and the right sides, and the sagittal MIP from each side was generated so that the MIP voxels from each side of the carotids could be extracted separately. As opposed to generating the sagittal MIP using the whole image, this approach increases the number of applicable voxels and allows to check the consistency of the IDIF peak timing between the two sides as a quality control measure. Voxels that best represent the input function within the carotids and are least contaminated from surrounding tissues are identified by performing a search for voxels with values and temporal patterns that best agree with the three arterialized venous blood samples collected during the last 30 min of the scan. This search also rejects overestimated voxels due to Gibb’s overshooting artifacts (see Fig. 1b). The proposed voxel search is defined below as the minimization of the Residual Sum of Squares w.r.t. Venous samples (VRSS) with a STC:

$$\underset{j\in {{STC}}_{j}=1}{\mathrm{arg min}}{VRSS}_{j},\quad {\text{where}}\; {VRSS}_{j}=\sum_{t=30\,\min}^{t=60\,\min}{\left({\lambda }_{j,t}-{V}_{t}\right)}^{2}$$

$${STC}_{j}={S}_{j}\cdot {T}_{j}, {S}_{j}=\left\{\begin{array}{ll} 1, & {\lambda }_{j,t\in1{st}\min}>{\text{threshold}}\\ 0, & {\text{otherwise}}\end{array}\right.$$

5 $${T}_{j}=\left\{\begin{array}{ll} 1, & {\text{voxel}}\; j\; {\text{with}}\; t_{p}\in PTW\\ 0, & {\text{otherwise}}\end{array}\right.$$

where *λ*_*j*,*t*_ is the sagittal MIP voxel *j* at time *t*. *V*_*t*_ is the Venous sample collected at time *t*. *STC*_*j*_ is the SpatioTemporal Constraint mask defined by the spatial mask *S*_*j*_ in the sagittal MIP and the temporal mask *T*_*j*_. The voxel intensity threshold for *S*_*j*_ is defined such that the width of the carotid spatial mask is 2–3 voxels (i.e. 50–60% of the physical diameter of the carotid artery) to exclude voxels near edges which are highly affected by PVE. A seed was placed at the location of the carotids in the spatial mask after the intensity thresholding in the sagittal MIP space to avoid the inclusion of structures outside the carotids with similar MIP intensity (e.g. sigmoid sinus). *t*_*p*_ is the time corresponding to the temporal signal peak. *PTW* is the Peak Timing Window defined by: (i) comparing *t*_*p*_ from the hottest cluster of voxels (>5 voxels) within *S*_*j*_ between the two sides’ sagittal MIPs, (ii) increasing the number of voxels within cluster until *t*_*p*_ from each side of carotids agrees with one another; if *t*_*p*_ from each side of the carotids does not agree after including all voxels within *S*_*j*_, the average *t*_*p*_ between the two sides is used as the resultant *t*_*p*_, and finally (iii) *PTW* is defined as the resultant *t*_*p*_ frame ± 1 frame (a frame duration of 5 s was used in this work around the IDIF peak in order to obtain accurate and reliable activity concentration at low counts as will be described later). The PTW was used to reject contaminated voxels with incorrect peak timing such as voxels with (delayed) peak timing from venous blood.

Ideally, a single quantitative voxel within the carotid artery would be sufficient to generate an accurate and reliable IDIF if data were noise-free. However, in practice more than one voxel is needed since the de-noising is not perfect. Therefore, once the voxel that minimizes the VRSS is found, the voxel search is repeated with the previously found voxel(s) excluded from the search until the resultant IDIF obtained using all of the previously found voxels contains a stable shape and peak magnitude within STC (i.e. mean absolute % difference across TAC within the 1st minute changes by less than 1% after the inclusion of an additional voxel). A blood sample free approach was also investigated by using all voxels within STC after a simple exclusion to extract the IDIF. In short, the simple exclusion rejects (i) the hottest cluster (top 10%) in *S*_*j*_ and (ii) MIP voxels in *S*_*j*_ that do not correspond to signals from carotids at the end of the scan as hot voxels from outside the carotids can get projected within *S*_*j*_ due to changes in the tracer distribution/increase in brain uptake (see overlapping hot voxels in Fig. 6c). Note that these voxels would be automatically rejected by the VRSS score.

### Experimental setups and reconstructions

A 16-cm-diameter cylindrical contrast phantom with a 10-mm-diameter sphere was filled with a 4:1 sphere-to-background ratio and injected with a total activity of 1.5 mCi of [^18^F]FDG. The phantom was scanned on the GE SIGNA PET/MR inside the Head Neck Unit (HNU) coil for 15 min. The list-mode data were unlisted/framed into dynamic 4D TOF sinograms according to the temporal count distribution (ranging from 4 million to 140 million counts) formed by the dynamic framing protocol for ^11^C human subject scans used in our institution (i.e. 60 s × 4, 120 s × 3, 300 s × 8, 600 s × 1; shorter temporal frames are used to capture the fast changing initial tracer kinetics while longer frames are used once the tracer distribution becomes more stable).

The dynamic 4D TOF sinogram data were reconstructed using PSF-TOFOSEM with and without the standard 3.5 mm FWHM transaxial and 1-4-1 axial filter, PSF-TOFBSREM with 8 different beta values ranging from 50 to 400 with an increment of 50, and PSF-HYPR4D-K-TOFOSEM with a 4D kernel size of 13 × 13 × 7 × 13 doxels (i.e. dynamic voxels or voxels with a 4th dimension in time) which corresponds to 5.6 mm FWHM in the spatial domain and 4 frames FWHM in the temporal domain. The 4D kernel size used in PSF-HYPR4D-K-TOFOSEM was selected to achieve sufficient 4D noise reduction without making the kernel matrix excessively non-sparse; i.e. the computation speed for the 4D kernel operations with this kernel size is 4 times faster than that of TOF projection operations and is thus making the HYPR4D kernel method practical in realistic scanning situations.

All reconstruction methods were run up to 10 iterations with 28 subsets except PSF-TOFBSREM which uses a GE pre-defined reconstruction protocol. For each beta value used in PSF-TOFBSREM, 2 iterations of PSF-OSEM, 3 iterations of PSF-BSREM, and 8 iterations of PSF-TOFBSREM were used sequentially according to the GE protocol for PSF-TOFBSREM. All corrections such as normalization, scatter, randoms, and CT-based attenuation correction of the phantom were applied for all reconstruction methods. The reconstructed image matrix size is 256 × 256 × 89 with voxel size of 1.39 × 1.39 × 2.78 mm^3^ for all methods. For each reconstruction method, the average Contrast Recovery Coefficient (CRC) ± STD (error bar) over all dynamic frames for the 10 mm sphere was computed and plotted as a function of average % voxel noise (voxel STD/mean*100) within the uniform background regions with the same size as the hot sphere as described in the NEMA protocol [13]. 3D volumes-of-interest (VOIs) were used instead of 2D regions-of-interest (ROIs) to improve the robustness of the CRC estimates.

**Fig. 2:**
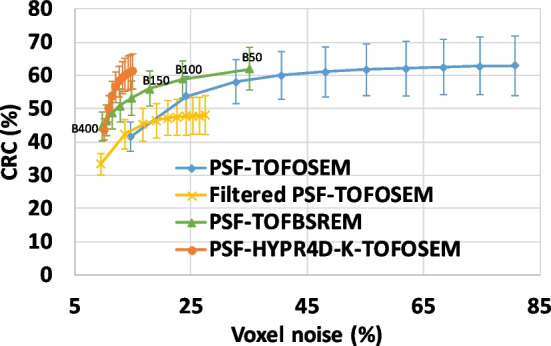
Contrast recovery coefficient versus voxel noise for the 10 mm sphere obtained from various reconstruction methods. Each point represents an OSEM iteration except for PSF-TOFBSREM where each point represents a beta value ranging from 50 to 400. The beta value increases from right to left with an increment of 50 (see labels for guidance) while the number of OSEM iteration increases from left to right

Human [^11^C]Raclopride (RAC), [^11^C]Dihydrotetrabenazine (DTBZ), and [^18^F]Fluorodeoxyglucose (FDG) scans (healthy controls), with 10 mCi bolus injection for ^11^C tracers and 5 mCi bolus injection for the ^18^F tracer (all with an injection pump), were acquired for 60 min on the GE SIGNA PET/MR. The data for the tracers used in this work were collected within the scope described in [14]. List-mode data were framed according to our standard dynamic framing protocol: 60 s × 4, 120 s × 3, 300 s × 8, 600 s × 1. The dynamic 4D TOF sinogram data were reconstructed using 4 iterations of PSF-TOFOSEM with and without the standard 3.5 mm FWHM transaxial and 1-4-1 axial post filter, PSF-TOFBSREM with various beta values, and 10 iterations of PSF-HYPR4D-K-TOFOSEM with the same 4D kernel size as mentioned above. The number of iterations selected for each method was based on the CRC versus noise trade-off (see Fig. 2) except for PSF-TOFBSREM which was run according to the GE protocol.

All corrections were applied, and Zero-echo Time (ZTE)-based MRAC was used for the attenuation correction of human subjects. TACs, image profile, and visual image quality comparisons were performed for the human scans. In addition, the IDIF based on the proposed voxel search method was extracted from the human [^18^F]FDG images and compared across all aforementioned reconstruction methods. For the IDIF extraction, the temporal framing within the first minute of the list-mode data (with the rest of the frame durations to be the same as above) was defined as 20 s × 1 and 5 s × 8 so that each temporal frame contained more than 0.8 million non-randoms counts. This limit was dictated by the observation that an underestimation error in the scatter correction was consistently present (data not shown) as was the error observed in estimated CRC presented in Table 1 at very low counts.

**Table 1:** Average voxel %CRC ± STD over 20 voxels within the 10 mm sphere across various count levels using the proposed voxel search for different reconstructions

Recon method\Avg. counts in TOF bin over angular views within target structure	2	3	4	5	6	7	8
PSF-TOFOSEM	41 ± 77	111 ± 27	97 ± 30	98 ± 33	113 ± 42	98 ± 28	98 ± 22
Filtered PSF-TOFOSEM	47 ± 31	90 ± 15	78 ± 10	75 ± 11	93 ± 17	80 ± 11	77 ± 10
PSF-TOFBSREM beta150	58 ± 66	112 ± 22	97 ± 19	93 ± 14	109 ± 23	97 ± 16	93 ± 13
PSF-TOFBSREM beta350	52 ± 29	89 ± 15	77 ± 11	75 ± 9	89 ± 15	80 ± 10	75 ± 9
PSF-HYPR4D-K-TOFOSEM	**72 ± 35**	**105 ± 16**	**98 ± 16**	**100 ± 16**	**103 ± 18**	**100 ± 18**	**98 ± 17**

The bold values show the best performance across count levels

Serial arterialized venous blood sampling was performed manually during the entire 60 min of [^18^F]FDG acquisition. The three samples collected during the last 30 min of the scan were linearly interpolated into 5 time points corresponding to the mid-points of the last 5 dynamic frames, which were then used to compute VRSS for the proposed voxel search method. The rest of the samples were used to validate the extracted IDIFs from all methods. Only samples taken after 10 min were included in the validation since only after that time the venous samples are considered equivalent to the arterial samples for [^18^F]FDG [11, 15]. Although arterial samples would provide a more stringent validation of the IDIF method as early peak values could be used as reference, our clinical protocol did not allow for collection of such samples.

To evaluate the accuracy of the proposed voxel search with a known activity concentration across different count levels, the proposed voxel search for the IDIF extraction was adapted for the contrast phantom study. The average number of counts in TOF sinogram bin within the high contrast target structure was estimated by first identifying the max counts along the radial TOF bins for each angular view, and the average number of (max) counts over all 224 angular views was computed as a measure of count level. As will be seen in the results, at least 20 voxels are needed to achieve a stable IDIF shape and peak for the proposed method. Instead of searching for voxels that best represent the measured blood samples, we searched for 20 voxels (out of a total of 87 within the 3D volume of the sphere; MIP was not applied since there would be <10 voxels left after MIP) that best represent the correct activity concentration within the 10 mm sphere (i.e. CRC = 100%). The well-defined 3D volume of the sphere was used instead of the proposed spatiotemporal constraint for the phantom study. The last 5 frames of the dynamic phantom images were used to compute the RSS score in CRC analogous to the VRSS in concentration for the human case, and 20 voxels with the lowest RSS score were searched for all reconstruction methods. The average voxel CRC ± STD over 20 voxels within the 10 mm sphere was tabulated for frames with different count levels. The reconstruction settings for all methods were the same as those used for the human studies.

Repeatability/intra-subject and inter-subject variability studies were conducted for the proposed reconstruction + IDIF method. One subject was scanned twice (8 months apart), and 10 different subjects were scanned with [^18^F]FDG on the GE SIGNA PET/MR using the same imaging protocol described above. For multiple scan comparisons, the activity concentration was converted to the Standard Uptake Value (SUV) to account for the injected dose and subject weight. The average IDIF as well as the measured venous samples (±STD) were computed over 10 subjects; both IDIF and venous blood measurements were resampled to 1 s time grid for all subjects before computing the average and STD. Time zero for all scans was defined by the specified count rate trigger; i.e. all scans started when the measured count rate within the field of view of the scanner exceeded 150,000 cps (>100 times higher than the background count rate).

## Results

### Regional CRC versus noise comparison

The CRC versus voxel noise comparison for the 10-mm-diameter sphere is shown in Fig. 2. PSF-TOFOSEM exhibited the highest noise increment per iteration compared to all other methods while the filtered PSF-TOFOSEM achieved noise reduction at the cost of lower CRC as expected. PSF-TOFBSREM achieved better CRC versus noise trajectory than PSF-TOFOSEM with and without filter. PSF-HYPR4D-K-TOFOSEM had the lowest noise increment per iteration and achieved even better CRC versus noise trade-off than PSF-TOFBSREM.

It can be observed that PSF-TOFBSREM with different beta values introduced different level of PVE as demonstrated by different CRC values. PSF-TOFBSREM with a high beta value (e.g. beta = 400) was observed to have similar contrast versus noise trade-off but with higher variation in CRC (i.e. bigger error bar) as compared to the early iteration estimate of PSF-HYPR4D-K-TOFOSEM. Additionally, 10 iterations of PSF-HYPR4D-K-TOFOSEM had similar noise level as compared to 1 iteration of PSF-TOFOSEM (which was used as the input image estimate for PSF-HYPR4D-K-TOFOSEM) but with CRC similar to that of the later iterations of PSF-TOFOSEM which exhibited a much higher noise level.

### Voxel CRC analysis within 10 mm sphere

The average voxel CRC ± STD over 20 voxels within the 10 mm sphere across various count levels using the proposed voxel search is tabulated in Table 1 for each reconstruction method. One can observe that much more accurate CRC can be obtained for all methods using the proposed voxel search as compared to using the VOI with the physical dimension of the sphere (as shown in Fig. 2) except at very low count level (e.g. count level 2 or 2 counts in TOF bin). This was likely due to the rejection of suboptimal voxels within the sphere when the reference activity concentration was known. At a relatively high count level (e.g. count level 8), the CRC estimate from the proposed PSF-HYPR4D-K-TOFOSEM was in agreement with the one obtained from PSF-TOFOSEM. For methods that do not preserve contrast well (e.g. filtered PSF-TOFOSEM and PSF-TOFBSREM with high beta values), the CRC using the voxel search was substantially lower than those that preserve contrast well as all voxel concentrations were underestimated within the sphere.

The proposed PSF-HYPR4D-K-TOFOSEM also showed the most accurate and consistent CRC estimates across all count levels compared to other methods. Interestingly, the STD from the proposed method was consistent across count levels as well (except at very low counts). This indicated that the voxel variation was not noise-induced and suggested that the proposed reconstruction method was able to make Gibb’s artifacts more predictable than other methods. Although filtered PSF-TOFOSEM and PSF-TOFBSREM showed lower STD than the proposed method, it was likely due to oversmoothing.

Another interesting observation was that at very low count level (e.g. level 2), the number of voxels with underestimated activity concentration outweighed that of overestimated concentration within the sphere for all methods. Consequently, an underestimation bias was observed for all methods with the proposed reconstruction method being the least biased. In addition, voxels with negative CRC were observed from PSF-TOFOSEM and PSF-TOFBSREM with low beta values within the sphere at very low count level (a noise induced behavior that is physically impossible as no voxel within the sphere should contain concentration lower than that of background outside the sphere). Although the use of MIP would reduce the underestimation, it is recommended to frame the data so that there are enough counts to obtain a reliable activity concentration estimate. Note that the frames near the IDIF peak used in this work had a count level of 3.

### TAC comparison

**Fig. 3:**
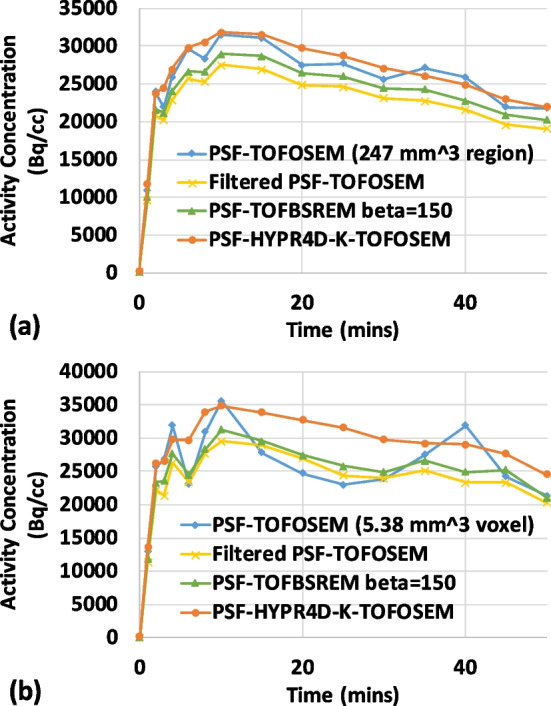
**a** Regional-level TAC and **b** voxel-level TAC comparisons in the caudate of the human [^11^C]RAC scan reconstructed using various methods

The TAC comparison for a relatively small region (247 mm^3^) placed in the caudate and for a single voxel within that region obtained from the human [^11^C]RAC scan is depicted in Fig. 3. At both regional and voxel levels, PSF-HYPR4D-K-TOFOSEM showed the lowest temporal noise as compared to other methods; e.g. the residual sum of squares from the model fitting (SRTM2 [16]) was ~3500 for the proposed PSF-HYPR4D-K-TOFOSEM, ~10,000 for filtered PSF-TOFOSEM, ~12,000 for PSF-BSREM with beta = 150, and ~56,000 for PSF-TOFOSEM in the caudate at voxel level. A fairly consistent difference in regional activity concentration over time (i.e. temporal pattern) between PSF-TOFOSEM and filtered PSF-TOFOSEM can be observed in Fig. 3a. This difference is likely due to the consistent PVE introduced by the post filter since the contrast in this region remains fairly constant over time for this tracer during the scan.

However, this difference was not consistently observed at the voxel level as shown in Fig. 3b likely due to the relatively high voxel noise in the PSF-TOFOSEM images. Both regional and voxel TACs obtained from PSF-HYPR4D-K-TOFOSEM showed similar overall difference in magnitude when compared to those obtained from filtered PSF-TOFOSEM. TACs obtained from PSF-TOFBSREM with beta = 150 were observed to have a slightly higher magnitude than those from filtered PSF-TOFOSEM but with similar noise-induced temporal patterns. Higher beta values resulted in lower magnitudes in TACs as expected (not shown).

### Image quality comparison

**Fig. 4:**
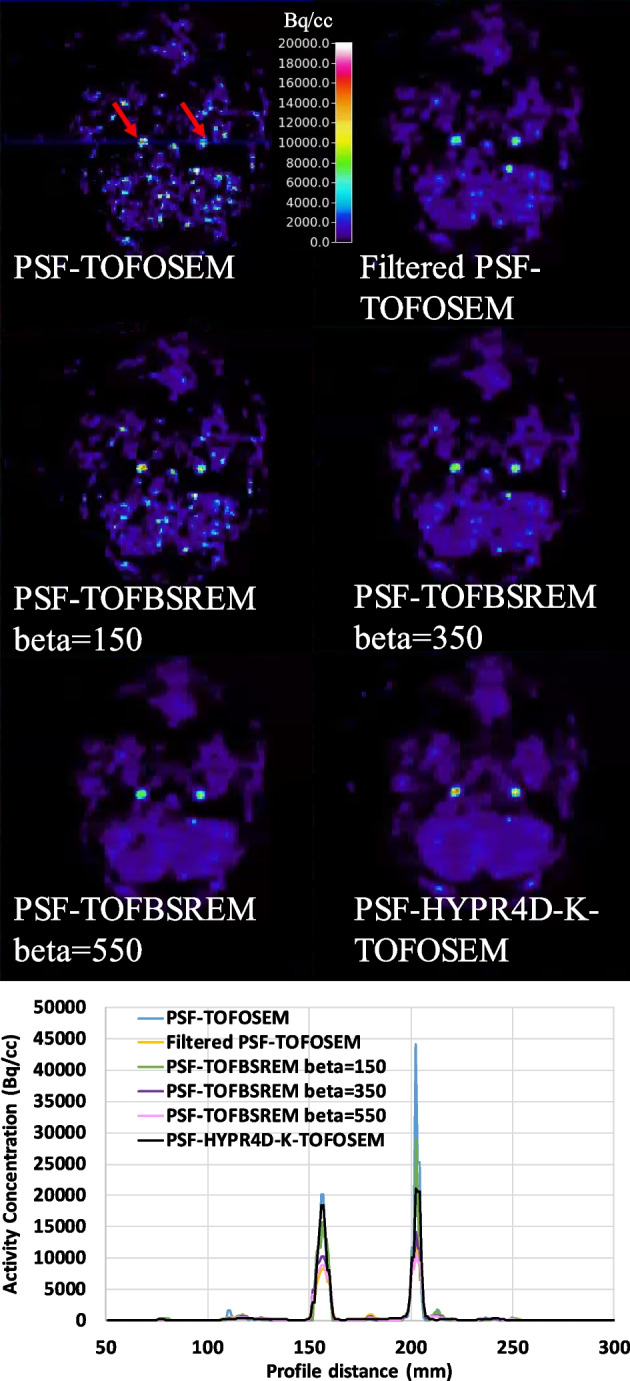
(Top) A transaxial slice which contains very high contrast (much greater than 4:1 contrast ratio used for the phantom) in the carotid arteries with a size of 5–7 mm in diameter (see red arrows) and (Bottom) line profile across the carotid arteries in a low count frame (1 min duration) of human [^11^C]DTBZ scan reconstructed using various methods. The location of the line profile can be seen from the PSF-TOFOSEM panel. Note that a substantially higher peak signal likely induced by noise can be observed from the right carotid artery than that from the left carotid in the PSF-TOFOSEM image, though the ground truth is not known here

**Fig. 5:**
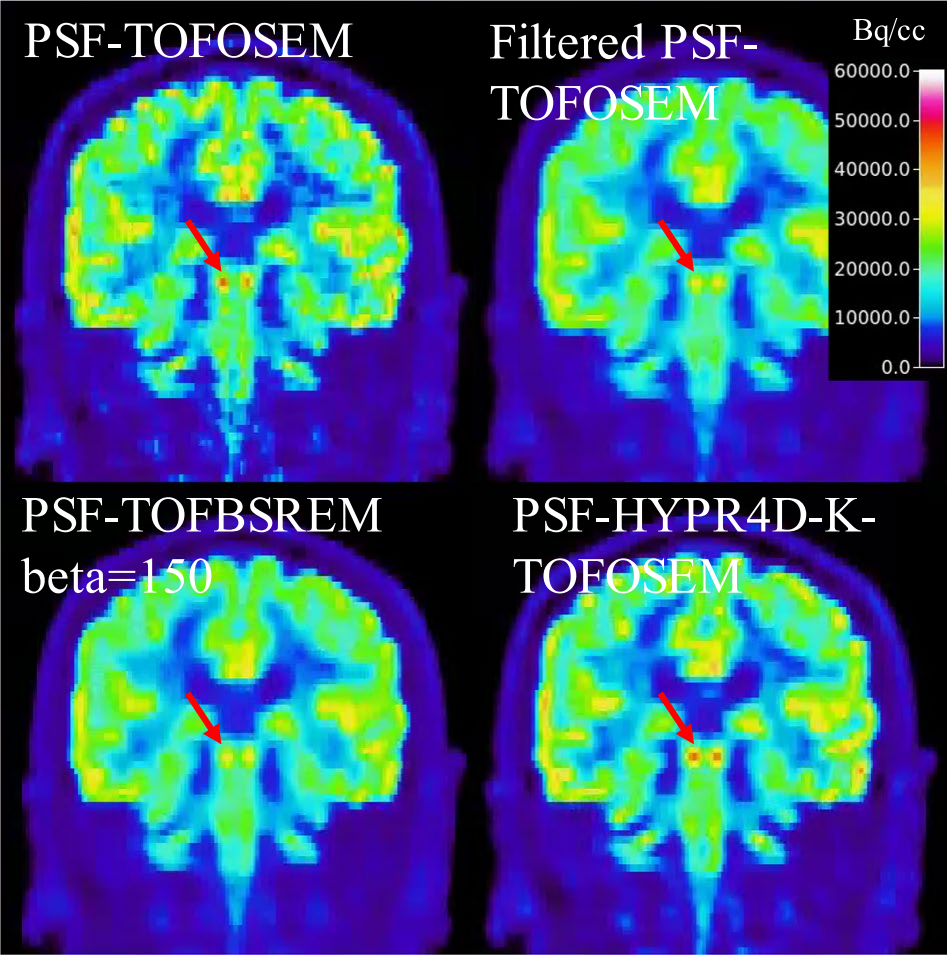
A coronal slice which contains colliculi with a size of ~ 4 mm in diameter (see red arrows) in a high count frame of human [^18^F]FDG (50 min post injection with 10 min frame duration) reconstructed using various methods. PSF-TOFBSREM with higher beta values were omitted since higher beta values only reduce contrast further without providing any benefit for high count data

We selected two relevant and challenging image quality test situations: a low count frame which contains very high contrast signal in the carotid arteries (5–7 mm in diameter) from a human [^11^C]DTBZ scan and a high count frame which contains moderate contrast in the colliculi (~4 mm in diameter) from a human [^18^F]FDG scan as shown in Figs. 4 and 5, respectively. As expected, post filter drastically reduced the contrast in the small structures especially when the structures had a very high contrast with respect to the surrounding tissues as shown in the line profile comparison in Fig. 4 (bottom); i.e. surrounding voxels contain much lower activity concentration values. The location of the line profile can be seen from the PSF-TOFOSEM panel in Fig. 4 (top). The additional PVE introduced by the post filter became stronger with increasing contrast and/or decreasing size of the target structures; the contrast was altered by the post filter more when the contrast was higher in general.

Interestingly, the reverse trend was observed for PSF-TOFBSREM; the contrast was altered less when the contrast was higher. For a given beta value (see beta = 150 for example) PSF-TOFBSREM was observed to preserve the contrast in small structure with very high contrast substantially better than PSF-TOFOSEM with post filter as shown in Fig. 4 (bottom) though the noise reduction was not sufficient for low count data with beta = 150. However, for moderate contrast levels such as what was observed in the colliculi (Fig. 5), the preservation of contrast for PSF-TOFBSREM with beta = 150 became similar to that of PSF-TOFOSEM with post filter as depicted by the similar magnitudes along TACs in Fig. 3 and similar contrast in small structures in Fig. 5 between them.

On the other hand, the preservation of contrast for PSF-HYPR4D-K-TOFOSEM showed less dependency on the contrast level and size of the target structures as compared to other regularization/noise reduction methods (i.e. more robust) as shown by the consistent performance observed when imaging the carotids with DTBZ or the colliculi with FDG (Figs. 4 and 5). Additionally, an asymmetric signal pattern (i.e. one peak was >200% higher with respect to the other peak) was observed between the two sides of carotids in PSF-TOFOSEM and PSF-TOFBSREM with low beta values (e.g. beta = 150) images, whereas all other methods showed a more symmetric pattern as depicted in Fig. 4 (bottom). The asymmetric pattern was considered to be likely noise-induced as a symmetric pattern would be expected for a healthy control.

### IDIF comparison

**Fig. 6:**
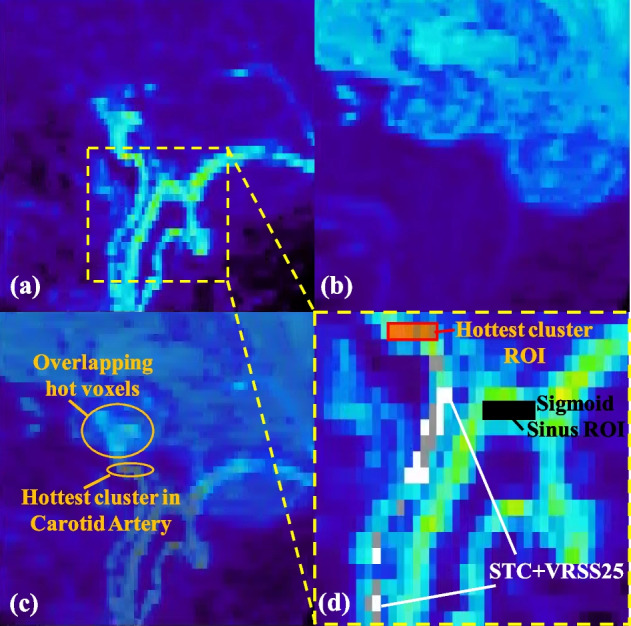
One of the two side sagittal MIPs reconstructed using the proposed PSF-HYPR4D-K-TOFOSEM from **a** the first 1 min of the FDG scan, **b** the last frame (50–60 min), **c** fusion image of (**a**) and (**b**) indicating the voxels excluded by simple exclusion, and **d** zoomed in sagittal MIP focusing on the ROI’s used in the analyses (note that STC + VRSS50 voxels are indicated by STC + VRSS25 voxels plus the grey voxels)

**Fig. 7:**
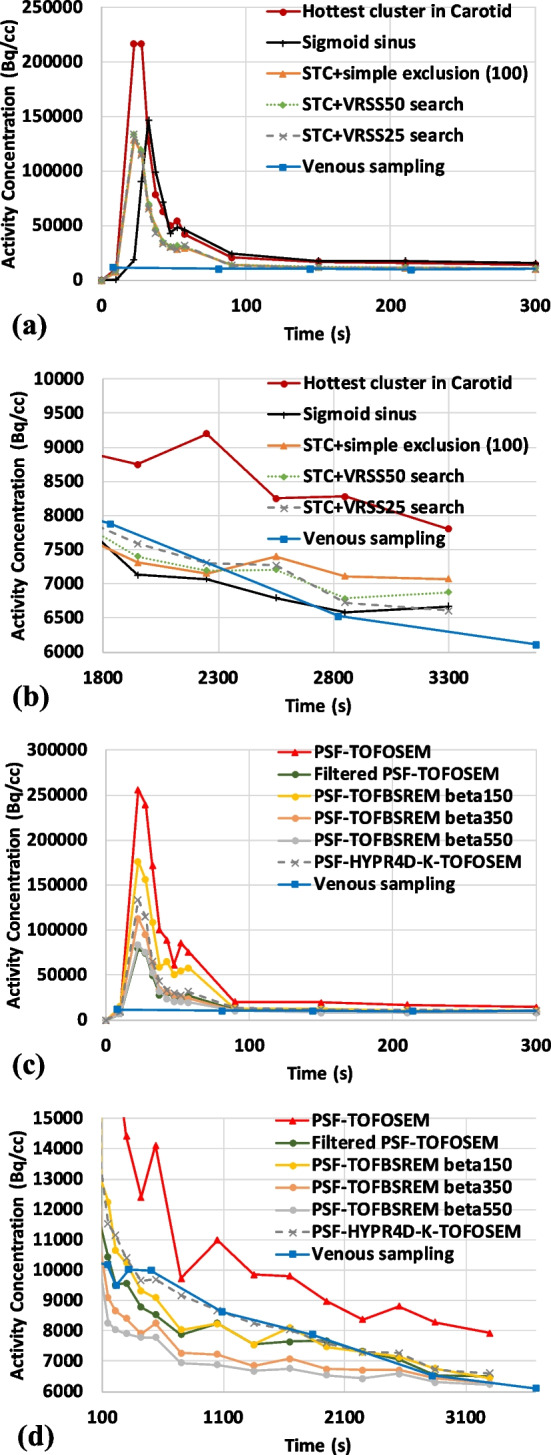
TACs extracted from the proposed PSF-HYPR4D-K-TOFOSEM + MIP-based voxel search and venous sampling for **a** the first 5 min of the scan, **b** the last 30 min of the scan, **c** the first 5 min using STC + VRSS25 with various reconstructions, and **d** after the IDIF peak to highlight the difference between curves from various reconstructions

Using the MIP voxels/ROIs defined in Fig. 6, the extracted TACs/IDIFs are depicted in Fig. 7. In this case, 100 voxels were found within the STC mask after the simple exclusion of voxels; i.e. excluding the overlapping hot voxels between the first and last frame as well as the hottest cluster of voxels as shown in Fig. 6c. It was observed that at least 20 MIP voxels were needed to obtain a stable shape and peak for the STC+VRSS based methods. Consequently, 50 and 25 voxels that best represent the three blood samples measured during the last 30 min of the scan were searched (i.e. VRSS50 and VRSS25, respectively) to investigate the effect of including more voxels on the VRSS score. As depicted in Fig. 7a, all of the STC based methods produced very similar shape and peak magnitude when more than 20 voxels were used to extract the IDIF. However, as the number of voxels increases the VRSS becomes worse (Fig. 7b).

The hottest cluster produced the highest peak magnitude, which was most likely overestimated as it was outside the typically observed range [17], while those from the other voxel search methods were within range. Moreover, the hottest cluster within the carotids does not produce an IDIF that matches well with blood samples likely due to the fact that the hottest cluster consists of mostly Gibb’s/overshoot artifact (overestimated) voxels from the PSF based reconstruction. As expected, STC+VRSS25 produced the best agreement with the blood samples during the last 30 min as shown in Fig. 7b. The reduction in VRSS from the last 5 frames achieved by VRSS25 (VRSS = 0.3 × 10^6^) is 97% compared to using the hottest cluster (VRSS = 12 × 10^6^), and 80% compared to the blood sample free method (i.e. STC + simple exclusion; VRSS = 1.4 × 10^6^). In addition, a 5 s temporal resolution was found to be able to resolve the difference in peak timing between carotid artery and sigmoid sinus; the peak from the manual venous sampling was completely missed in this case likely due to manual sampling error (Fig. 7a).

When comparing the input function extracted using STC+VRSS25 between different reconstructions as shown in Fig. 7c and d, the highest peak magnitude was obtained from the PSF-TOFOSEM due to the fact that the images were filled with noise-induced hot voxels and noise-induced high concentration values within carotids (e.g. see Fig. 4 top-left panel and bottom line profile). Similarly, the later part of IDIF obtained from PSF-TOFOSEM was overestimated compared to the measured blood samples as well likely due to the noise-induced magnitude and noise-amplified Gibb’s overshooting artifacts. As a result, the worst VRSS score was obtained from PSF-TOFOSEM compared to all other methods. On the other hand, the IDIF peak magnitude from filtered PSF-TOFOSEM was most likely underestimated due to the additional PVE introduced by the post filter for such a small structure with high contrast as was observed previously in Fig. 4 with a different tracer. Interestingly, filtered PSF-TOFOSEM performed very well near the end of scan when the contrast vanished within the carotids; however, during earlier time points the activity concentrations were underestimated compared to blood samples likely due to the additional PVE from the post filter in the presence of contrast.

Similar to what was observed in Figs. 4 and 5, PSF-TOFBSREM/Q.Clear with beta = 150 showed a substantially higher IDIF peak magnitude than that from filtered PSF-TOFOSEM (i.e. when contrast was high), whereas they became very similar during the later part of IDIF as the contrast in activity concentration between the carotids and surrounding tissues decreased. Furthermore, it was observed that PSF-TOFBSREM with beta = 550 produced nearly identical IDIF peak magnitude and shape for the early part of IDIF as that from filtered PSF-TOFOSEM (see Fig. 7c); however, the activity concentration/preservation of contrast with beta = 550 became lower/worse than that from filtered PSF-TOFOSEM or blood samples during the later part of IDIF as shown in Fig. 7d. In general, PSF-TOFBSREM does not preserve the temporal pattern well when the contrast within the structure varies drastically over time.

The input function obtained from PSF-HYPR4D-K-TOFOSEM was observed to match with the blood sampling better than those obtained from other regularization methods thus demonstrating better preservation of contrast with less dependency on the contrast and size of the structure while achieving 4D noise reduction. PVE was effectively minimized by the proposed voxel search with spatiotemporal constraint and resolution modeling while rejecting Gibb’s overshoot voxels.

### Repeatability/intra-subject and inter-subject variability

**Fig. 8:**
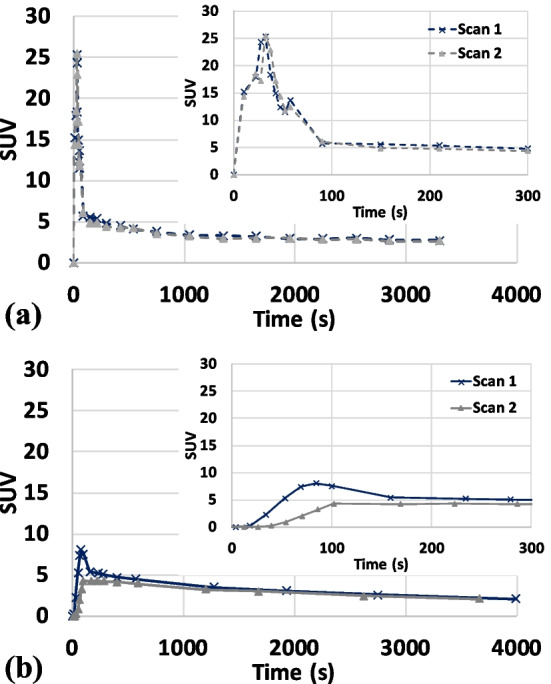
**a** IDIFs extracted from two scans of the same subject and **b** measured venous blood samples for the corresponding scans. Both plots include the whole curve as well as a zoomed-in focusing on the initial 300 s

**Fig. 9:**
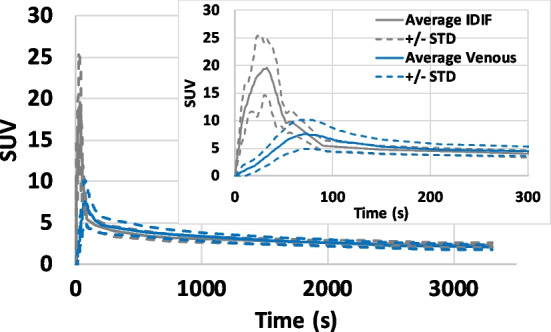
The average IDIF ± STD obtained from the proposed method and the average venous samples ± STD over 10 subjects

The IDIFs extracted from two scans of the same subject using the proposed method and the corresponding venous blood samples are depicted in Fig. 8a and b, respectively. One can observe that the IDIFs were similar in shape as well as in peak timing and magnitude. This demonstrated the effectiveness of the proposed STC. On the other hand, our measured venous blood samples showed much worse reproducibility during the initial 300 s than our IDIF estimates likely due to the inconsistent arterialization of the venous samples. The average IDIF ± STD obtained from the proposed method and the average venous samples ± STD over 10 subjects are shown in Fig. 9. It can be observed that the difference in peak timing between the average IDIF (peaked at ~35 s) and the average venous blood samples (peaked at ~74 s) was substantially greater than the width of our PTW. This indicated that the temporal constraint used in our voxel search was sufficient to reduce the contamination from voxels with peak timing of the venous blood. The average IDIF peak magnitude was ~20 ± 5 SUV while the average peak magnitude from the venous sampling was ~7.5 ± 2.5 SUV (delayed and dispersed w.r.t. IDIF, as expected).

## Discussion

This work presents the first application of HYPR4D kernel method on TOF PET data and demonstrates improvements in CRC versus noise trajectory, TACs, general image quality, and IDIF as compared to other TOF reconstructions/regularization methods that include PSF resolution modeling. The improvements are particularly relevant when imaging small structures. Due to the intrinsic data driven nature of the proposed method, spatiotemporal image features such as CRC and TAC can be preserved better than other methods while achieving effective 4D noise reduction (using CRC at high counts and TAC with sufficient number of voxels from PSF-TOFOSEM as the reference).

The proposed voxel search method for extracting an IDIF minimally impacted by PVE was built upon the excellent de-noising and preservation of spatiotemporal features provided by the proposed HYPR4D kernel method. The proposed method enables the use of a very limited number of voxels to obtain IDIF; as a result, strict voxel selection criteria can be applied. For instance, the use of PSF resolution modeling, MIP, and spatial mask/constraint corrects and minimizes the voxels with underestimated activity concentration within carotids while the VRSS score rejects the voxels with overestimated activity concentration due to overshooting/Gibb’s artifacts as well as contamination from surrounding tissues. In addition, the temporal constraint on the IDIF peak timing further reduces the contribution from surrounding tissues and noise. Consequently, the need of an explicit partial volume correction to obtain a quantitative IDIF is minimized as quantitatively accurate voxels already exist within the target structure in the image reconstructed with PSF resolution modeling.

As can be observed in Fig. 7c, there exists a beta value between 150 and 350 for PSF-TOFBSREM that would produce similar IDIF peak magnitude as PSF-HYPR4D-K-TOFOSEM. However, the later part of the IDIF with that beta value would not match well with the blood sample values. This can be inferred in Fig. 7d since the preservation of contrast worsens for PSF-TOFBSREM as the contrast between the target structure and surrounding tissues decreases. Similar behavior was observed using data from other tracers examined in this work. In addition, the differences in magnitude between peaks obtained from different reconstructions were observed to be higher in the human study than those in the phantom study (see count level 3 in Table 1) likely due to that (i) the underestimation in activity concentration was worse in the carotids than in the 10 mm sphere for methods that do not preserve contrast well (e.g. filtered PSF-TOFOSEM and PSF-TOFBSREM with high beta values) as the diameter of the carotids is approximately half the size of the sphere diameter; i.e. preservation of contrast gets worse for smaller structures, and (ii) the overestimation was worse/higher for methods with insufficient noise reduction (e.g. PSF-TOFOSEM and PSF-TOFBSREM with low beta values) since MIP is sensitive to high voxel variance/noise induced hot voxels.

A significant limitation in the validation of the IDIF extraction method was the lack of arterial samples. While the samples at later times are very similar, it is well known that the venous samples do not agree with the arterial samples initially; e.g. the peak signal from venous sampling is delayed and dispersed compared to arterial sampling [18]. Therefore, the peak magnitude of the IDIFs can only be compared to the literature values. Except for the methods that suffered strongly from noise-induced bias and/or Gibb’s artifacts, most methods produced peak magnitudes within the typically observed range. As a result, further validation is needed using a better gold standard (e.g. arterial samples or IDIF from the aorta [17]). Nevertheless, the trends/differences observed between reconstruction methods are consistent across all phantom and human studies. It is important to note that the proposed IDIF extraction method still requires metabolite correction in tracers where radiometabolites are present.

**Fig. 10:**
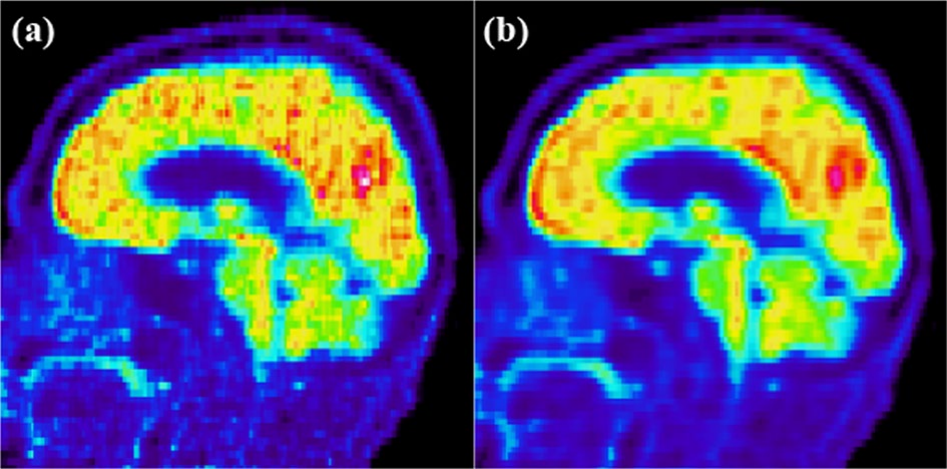
An example of the sagittal view of a human brain FDG uptake showing the vertical streaking artifacts in the **a** PSF-TOFOSEM image and the absence of the artifacts in the **b** PSF-HYPR4D-K-TOFOSEM image. The streaking artifacts are also visible in the TOF Q.Clear images with low beta values (not shown)

There is an additional advantage of using PSF-HYPR4-K-TOFOSEM. Recently, a hybrid-space PSF resolution modeling has been proposed to mitigate the striping/streaking artifacts introduced by the projection-based PSF model when reconstructing image with voxel size smaller than the width of projection lines of response [19]. Due to the convolutional nature of the proposed kernel matrix and that the high frequency features are updated in a cleaner fashion, the proposed PSF-HYPR4D-K-TOFOSEM intrinsically remedies the streaking artifacts in the image as shown in Fig. 10. Moreover, since the kernel matrix is de-coupled from the PSF model of the system, unlike in the hybrid-space PSF method our de-noising kernel size is not limited/restricted by the total PSF kernel of the system. As a result, better noise reduction performance can be achieved by the proposed method in 4D while mitigating streaking artifacts.

## Conclusion

The preservation of contrast for the proposed PSF-HYPR4D-K-TOFOSEM was observed to be better and less dependent on the contrast and size of the target structure as compared to other regularization methods such as PSF-TOFBSREM and PSF-TOFOSEM with filter. At the same contrast level, PSF-HYPR4D-K-TOFOSEM also achieved better 4D noise suppression than other methods. These promising results on TOF PET data demonstrated that the proposed HYPR4D kernel method is likely suitable for all imaging tasks without requiring any prior information and can further improve the effective sensitivity of the imaging system. In addition, the proposed reconstruction method enabled a new approach to define an image-derived input function that appears to minimally suffer from noise and PVE thus minimizing the need for an explicit partial volume correction.

## Data Availability

The phantom data are available upon request. The human data are not publicly available due to subject confidentiality.

## Abbreviations

BSREM: Block sequential regularized expectation maximization
cps: Counts per second
CRC: Contrast recovery coefficient
DTBZ: Dihydrotetrabenazine
FDG: Fluorodeoxyglucose
FWHM: Full width at half maximum
GE: General electronics
HNU: Head neck unit
HYPR: HighlY constrained backPRojection
HYPR4D: 4D modified HYPR
IDIF: Image-derived input function
LOR: Line of response
MIP: Maximum intensity projection
MR or MRI: Magnetic resonance imaging
MRAC: MR-based attenuation correction
NLM: Non-local mean
OSEM: Ordered subset expectation maximization
PET: Positron emission tomography
PSF: Point spread function
PTW: Peak timing window
PVE: Partial volume effect
RAC: Raclopride
ROI: Region of interest
RSS: Residual sum of squares
SRTM2: 2 Pass simplified reference tissue model
STC: SpatioTemporal Constraint
STD: Standard deviation
SUV: Standard uptake value
TAC: Time activity curve
TOF: Time of flight
VOI: Volume of interest
VRSS: RSS w.r.t. venous blood samples
ZTE: Zero-echo time

## References

[1] Wang G, Qi J (2015). PET image reconstruction using kernel method. IEEE Trans Med Imag.

[2] Bland J, Mehranian A, Belzunce MA, Ellis S, McGinnity CJ, Hammers A, Reader AJ (2018). MR-guided kernel EM reconstruction for reduced dose PET imaging. IEEE Trans Radiat Plasma Med Sci.

[3] Buades A, Coll B (2005). A non-local algorithm for image denoising. Comput Vis Pattern.

[4] Christian BT, Vandehey NT, Floberg JM, Mistretta CA (2010). Dynamic PET denoising with HYPR processing. J Nucl Med.

[5] Spencer B, Qi J, Badawi RD, Wang G (2017). Dynamic PET image reconstruction for parametric imaging using the HYPR kernel method. Proc SPIE Med Imag.

[6] Wang G (2019). High temporal-resolution dynamic PET image reconstruction using a new spatiotemporal kernel method. IEEE Trans Med Imag.

[7] Cheng J-CK, Bevington C, Rahmim A, Klyuzhin I, Matthews J, Boellaard R, Sossi V (2021). Dynamic PET image reconstruction utilizing intrinsic data-driven HYPR4D de-noising kernel. Med Phys.

[8] Grant AM, Deller TW, Khalighi MM, Maramraju SH, Delso G, Levin CS (2016). NEMA NU 2–2012 performance studies for the SiPM-based ToF-PET component of the GE SIGNA PET/MR system. Med Phys.

[9] Liberini V, Pizzuto DA, Messerli M, Orita E, Grünig H, Maurer A, Mader C, Husmann L, Deandreis D, Kotasidis F, Trinckauf J, Curioni A, Opitz I, Winklhofer S, Huellner MW (2022). BSREM for brain metastasis detection with 18F-FDG-PET/CT in lung cancer patients. J Digit Imaging.

[10] Tian D, Yang H, Li Y, Cui B, Lu J (2022). The effect of Q.Clear reconstruction on quantification and spatial resolution of 18F-FDG PET in simultaneous PET/MR. EJNMMI Phys.

[11] Zanotti-Fregonara P, Chen K, Liow J-S, Fujita M, Innis RB (2011). Image-derived input function for brain PET studies: many challenges and few opportunities. J Cereb Blood Flow Metab.

[12] Rahmim A, Qi J, Sossi V (2013). Resolution modeling in PET imaging: theory, practice, benefits, and pitfalls. Med Phys.

[13] NEMA. Performance measurements of positron emission tomographs, Rosslyn, VA. NEMA Standards Publication NU 2–2007, pp. 26–33; 2007.

[14] Mannheim JG, Cheng J-CK, Vafai N, Shahinfard E, English C, McKenzie J, Zhang J, Barlow L, Sossi V (2021). Cross-validation study between the HRRT and the PET component of the SIGNA PET/MRI system with focus on neuroimaging. EJNMMI Phys.

[15] van der Weerdt AP, Klein LJ, Visser CA, Visser FC, Lammertsma AA (2002). Use of arterialised venous instead of arterial blood for measurement of myocardial glucose metabolism during euglycaemic-hyperinsulinaemic clamping. Eur J Nucl Med.

[16] Wu Y, Carson R (2002). Noise reduction in the simplified reference tissue model for neuroreceptor functional imaging. J Cereb Blood Flow Metab.

[17] Viswanath V, Chitalia R, Pantel AR, Karp JS, Mankoff DA (2021). Analysis of 4D data for total-body PET imaging. PET Clin.

[18] http://www.turkupetcentre.net/petanalysis/input_venous.html.

[19] Deller TW, Ahn S, Jansen FP, Schramm G, Wangerin KA, Spangler-Bickell MG, Stearns CW, Khalighi MM. Implementation and image quality benefit of a hybrid-space PET point spread function. IEEE NSS-MIC Conference 2021, Yokohama, Japan.

